# Studies on the Interaction of the Histone Demethylase KDM5B with Tricarboxylic Acid Cycle Intermediates

**DOI:** 10.1016/j.jmb.2017.08.007

**Published:** 2017-09-15

**Authors:** Hanna Tarhonskaya, Radosław P. Nowak, Catrine Johansson, Aleksandra Szykowska, Anthony Tumber, Rebecca L. Hancock, Pauline Lang, Emily Flashman, Udo Oppermann, Christopher J. Schofield, Akane Kawamura

**Affiliations:** 1 Chemistry Research Laboratory, Department of Chemistry, University of Oxford, 12 Mansfield Road, Oxford, OX1 3TA, United Kingdom; 2 Structural Genomic Consortium, University of Oxford, Old Road Campus, Roosevelt Drive, Oxford, OX3 7DQ, United Kingdom; 3 Botnar Research Centre, NIHR Oxford Biomedical Research Unit, University of Oxford, Windmill Road, Oxford, OX3 7LD, United Kingdom

**Keywords:** 2OG, 2-oxoglutarate, 2HG, 2-hydroxyglutarate, KDM, lysine demethylase, TCA, tricarboxylic acid, JmjC, Jumonji C, MALDI-TOF-MS, matrix-assisted laser desorption/ionisation–time of flight–mass spectrometry, IF, immunofluorescence, DNA, deoxyribonucleic acid, FDH, formaldehyde dehydrogenase, FIH, hypoxia-inducible factor, epigenetics, lysine JmjC demethylase, 2-oxoglutarate oxygenase, TCA cycle inhibition, KDM5B

## Abstract

Methylation of lysine-4 of histone H3 (H3K4me_*n*_) is an important regulatory factor in eukaryotic transcription. Removal of the transcriptionally activating H3K4 methylation is catalyzed by histone demethylases, including the Jumonji C (JmjC) KDM5 subfamily. The JmjC KDMs are Fe(II) and 2-oxoglutarate (2OG)-dependent oxygenases, some of which are associated with cancer. Altered levels of tricarboxylic acid (TCA) cycle intermediates and the associated metabolites D- and L-2-hydroxyglutarate (2HG) can cause changes in chromatin methylation status. We report comprehensive biochemical, structural and cellular studies on the interaction of TCA cycle intermediates with KDM5B, which is a current medicinal chemistry target for cancer. The tested TCA intermediates were poor or moderate KDM5B inhibitors, except for oxaloacetate and succinate, which were shown to compete for binding with 2OG. D- and L-2HG were moderate inhibitors at levels that might be relevant in cancer cells bearing isocitrate dehydrogenase mutations. Crystallographic analyses with succinate, fumarate, L-malate, oxaloacetate, pyruvate and D- and L-2HG support the kinetic studies showing competition with 2OG. An unexpected binding mode for oxaloacetate was observed in which it coordinates the active site metal via its C-4 carboxylate rather than the C-1 carboxylate/C-2 keto groups. Studies employing immunofluorescence antibody-based assays reveal no changes in H3K4me_3_ levels in cells ectopically overexpressing KDM5B in response to dosing with TCA cycle metabolite pro-drug esters, suggesting that the high levels of cellular 2OG may preclude inhibition. The combined results reveal the potential for KDM5B inhibition by TCA cycle intermediates, but suggest that in cells, such inhibition will normally be effectively competed by 2OG.

## Highlights

• The JmjC histone demethylase KDM5B has roles in cell proliferation and tumor maintenance.
• Elevated TCA cycle metabolite levels may promote tumorigenesis by inhibition of JmjC KDMs.
• Crystallographic studies provide evidence for KDM5B interaction with TCA cycle metabolites.
• TCA cycle metabolites can inhibit KDM5B in a 2OG competitive manner.
• Oxaloacetate binds KDM5B chelating the active site metal via its C-4 carboxylate.

## Graphical abstract

**Figure:**
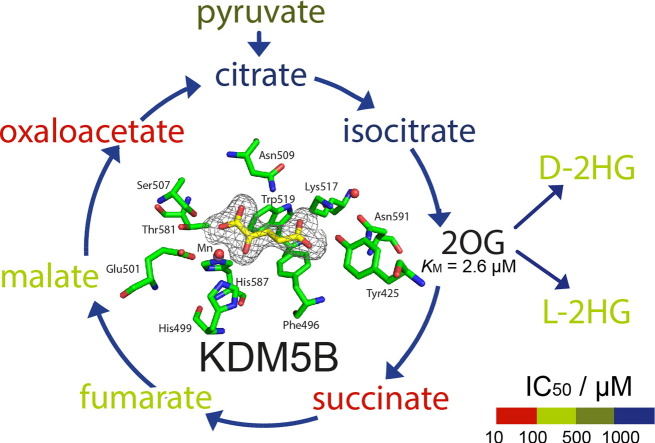

## Introduction

Dynamic changes in post-translational modifications to chromatin, in particular to the N-terminal tails of histone H3, contribute to the regulation of many cellular functions and modulate transcription in both healthy and diseased states [1], [2], [3]. Histone H3 lysine 4 (H3K4) methylation is an important modification that is normally activating with respect to transcription [4]. Removal of the H3K4 methylation regulates expression and is performed by histone demethylases from two different families [5]. The flavin adenine dinucleotide-dependent demethylases LSD1/2 (KDM1 family) specifically catalyze demethylation of di- and mono-methylated states of H3K4 (H3K4me_1_ and H3K4me_2_), while all methylated states of H3K4 (H3K4me_1_, H3K4me_2_ and H3K4me_3_) are substrates for the KDM5 family of demethylases, also known as the JARID1s [1], [6], [7]. The KDM5 family contains the Jumonji C (JmjC) domain and belongs to the superfamily of Fe(II)/2-oxoglutarate (2OG)-dependent oxygenases [8], [9]. The 2OG oxygenases use Fe(II) as a cofactor and 2OG as a co-substrate, conversion of which to succinate and CO_2_ is coupled to oxidation of methylated lysine residues in histone substrates to give hemiaminal intermediates, subsequent decomposition of which results in the formation of the corresponding demethylated lysine residue and formaldehyde [10].

The human KDM5 family comprises four isoforms (KDM5A-D), which in addition to the catalytic JmjC domain also contain an N-terminal Jumonji N-domain, a deoxyribonucleic acid (DNA)-binding ARID domain, a C5HC2 zinc finger, a PLU1-motif and two or three PHD domains [8], [11], [12]. KDM5 demethylation activity is involved in regulating cell differentiation and proliferation (KDM5A and KDM5B) and has been related to disease states, especially cancer [1], [13]. The KDM5 enzymes have roles in tumor initiation, maintenance/survival and development of drug resistance [13], [14]. KDM5A (JARID1A, RBP2) is a retinoblastoma RB-binding protein and mediates metastasis in estrogen receptor-negative breast cancers [15], while KDM5B (JARID1B or PLU-1) has roles in tumor maintenance [16], melanoma metastatic progression and resistance to cytotoxic agents and BRAF inhibitors [13]. KDM5C is involved in neuronal development [17], and KDM5D, which is encoded by a Y-chromosome-encoded (male specific) gene, has a role in spermatogenesis [18], Consequently, KDM5 members are current medicinal chemistry targets for cancer, with KDM5B being a particular focus due to its role in tumor maintenance and progression [13].

The JmjC demethylases employ 2OG as a co-substrate and have potential to be inhibited by structurally related tricarboxylic acid (TCA) cycle metabolites [19]. Previous work has shown inhibition of certain 2OG oxygenases by succinate, fumarate [20], [21], [22], [23], [24] or D-/L-2-hydroxyglutarate (D-/L-2HG) [25], [26], [27], including the histone demethylase KDM4A [28], [29]. These observations are of interest since mutations to genes encoding for succinate dehydrogenase or fumarate hydratase in cancer cells lead to the accumulation of succinate or fumarate, the metabolic changes that have been associated with tumorigenesis [30], [31], [32]. Elevated succinate and fumarate levels occur in paragangliomas, pheochromocytomas, gastric tumors (succinate) and hereditary leiomyomatosis (fumarate) [33], [34]. “Gain of function” mutations to genes encoding for isocitrate dehydrogenase 1 or 2 (IDH1/2) in various tumors and leukemia lead to accumulation of high levels of D-2HG [35], [36], [37]. It is proposed that accumulation of some TCA cycle metabolites/2HG promotes tumorigenesis by altering modifications to chromatin, including histone and DNA methylation patterns [34]. Accumulation of D-2HG in cells has been reported to affect the H3K9 and H3K27 methylation status [38], [39], and succinate dehydrogenase/fumarate hydratase mutant tumors have been linked to global increases in DNA methylation/hydroxymethylation levels [40], [41]. Thus, the effects of TCA cycle metabolites on the levels of other histone methylation marks, including transcriptionally important H3K4 methylation, merit detailed investigation.

We report studies on the enzyme activity inhibition of isolated KDM5B, a current medicinal chemistry target, by TCA cycle metabolites and D- and L-2HG. Kinetic studies revealed a large difference in the extent of inhibition, with oxaloacetate and succinate being the most potent of the tested compounds. Crystallographic analyses support the findings suggesting competition of TCA cycle intermediates with 2OG and reveal an unexpected coordination mode for oxaloacetate. In contrast with some of the other 2OG oxygenases acting on chromatin, cellular studies on the effects of elevated TCA cycle metabolites on H3K4 methylation imply that the relatively high 2OG levels may “outcompete” the elevated TCA intermediate levels.

## Results

### *In vitro* biochemical studies show inhibition of KDM5B by succinate and oxaloacetate

We initially investigated inhibition of purified recombinant KDM5B (produced in Sf9 insect cells, M1-R822) by TCA cycle metabolites. KDM5B catalyzes demethylation of H3K4me_3_, H3K4me_2_ and (with lower activity) H3K4me_1_; in line with the previous work, the H3K4me_2_(1–21) peptide fragment was chosen for inhibition studies to avoid complications due to sequential demethylation that is observed when using the analogous H3K4me_3_ peptide [8]. Previous kinetic studies on KDM5B and H3K4me_2_ were conducted using a formaldehyde dehydrogenase (FDH)-coupled assay using 15-mer and 21-mer H3 peptide fragments, and recombinant truncated KDM5B constructs, without the PLU1, PHD2 and PHD3 domains (KDM5B(1–769) or KDM5B(1–822) [8], [42]). Reported *K*_m_^app^(2OG) and *K*_m_^app^(H3K4me_2_(1–15)) were 6 and 3.6 μM, respectively [42], whereas the *K*_m_^app^ for H3K4me_2_(1–21) was 0.85 μM [8]. The differences in the reported *K*_m_^app^ values may be attributed, at least in part, to different lengths of the peptide substrates as precedented in work with other 2OG oxygenases [43], [44], [45].

We used matrix-assisted laser desorption/ionization–time of flight–mass spectrometry (MALDI-TOF-MS) for direct analysis of methylated and demethylated peptide ratios (by analyzing peak intensities), to avoid the possibility of FDH inhibition which could interfere with results when using the FDH-coupled enzyme assay. As a prelude to the inhibition studies, *K*_m_^app^ values for 2OG and H3K4me_2_(1–21) peptide were determined for KDM5B(1–822) using MALDI-TOF-MS to identify appropriate experimental conditions for inhibition studies. Our determined values were *K*_m_^app^(2OG) = 2.6 ± 0.6 μM, *K*_m_^app^(H3K4me_2_(1–21)) was ~ 0.5 μM and the *k*_cat_^app^ was 0.015 ± 0.01 s^− 1^ (Supplementary Information, Fig. S1). Thus, the results of our *K*_m_^app^(H3K4me_2_(1–21)) determination are in agreement with the previously reported FDH-based results [8]. The *K*_m_^app^(H3K4me_2_) for the 21-mer peptide was lower than the MALDI-TOF-MS limit of detection, which prevented a more accurate determination.

The inhibition of TCA cycle metabolites was then assessed by determining the IC_50_ values with the 2OG concentration fixed at the *K*_m_^app^(2OG) level (3 μM) and saturating levels of H3K4me_2_(1–21) peptide substrate (5 μM). The highest levels of inhibition were observed in the presence of oxaloacetate (IC_50_ 15 ± 10 μM; Table 1, Supplementary Information Fig. S2) and succinate (IC_50_ 62 ± 19 μM; Table 1). For pyruvate, citrate and isocitrate, millimolar levels were required to observe inhibition (if any) (IC_50_ 703 ± 26 μM for pyruvate, > 1 mM for citrate/isocitrate). D- and L-2HG inhibited KDM5B-catalyzed demethylation with IC_50_ values in a range of 150–200 μM, that is, less potently than for some other reported histone demethylases (e.g., KDM4A, the IC_50_ values for both D- and L-2HG ~ 25 μM) [28]; these values, however, do not exclude inhibition in tumor cells where D-2HG levels can reach the mM range [46].

**Table 1:** Inhibition of KDM5B by TCA cycle intermediates

Compound	IC_50_ (μM)
Succinate	62 ± 19
Fumarate	210 ± 80
D-2HG	203 ± 90
L-2HG	150 ± 40
Malate	127 ± 72
Citrate	Inactive (18% inhibition at 1 mM)
Pyruvate	703 ± 26
Oxaloacetate	15 ± 10
Isocitrate	Inactive (10% inhibition at 1 mM)

IC_50_ values for KDM5B by TCA cycle metabolites. Assay conditions: 0.6 μM KDM5B, 3 μM 2OG, 5 μM H3K4me_2_(1–21), 10 μM Fe(II), 500 μM L-ascorbate in 50 mM Hepes, 50 mM NaCl (pH 7.5). Errors represent 95% confidence intervals. The levels of methylated and demethylated peptides were analyzed using MALDI-TOF-MS.

**Fig. 1:**
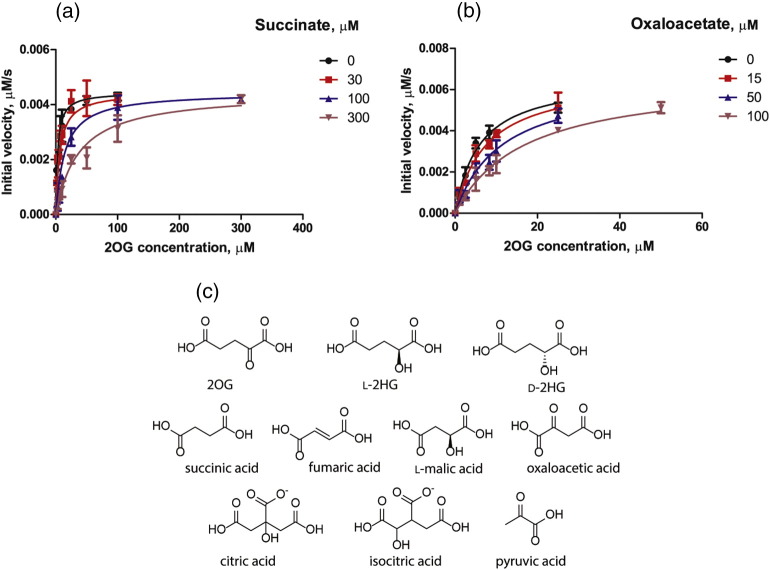
Determination of *K*_i_ values for inhibition of KDM5B by succinate (a) and oxaloacetate (b). Assay conditions: 0.6 μM KDM5B was incubated with various concentrations of 2OG, 5 μM H3K4me_2_(1–21), 10 μM Fe(II), and 500 μM L-ascorbate and different concentrations of succinate (0, 30, 100 and 300 μM) or oxaloacetate (0, 15, 50 and 100 μM), buffer: 50 mM Hepes, 50 mM NaCl (pH 7.5). Error bars represent the SD for triplicate assays. The levels of methylated and demethylated peptide were analyzed using MALDI-TOF-MS. Analysis of data was performed using GraphPad Prism 5.0 (Supplementary Information, Tables S1–S3). C. Structures of the TCA cycle intermediates investigated as KDM5B inhibitors.

We then determined inhibitory constants (*K*_i_) for succinate and oxaloacetate using the MALDI-TOF-MS assay with respect to 2OG. *K*_m_^app^ for 2OG was assessed in the presence of different concentrations of succinate and oxaloacetate (Fig. 1, Table 2); the data fitted best to a competitive inhibition model using non-linear regression (more details are given in the Supplementary Information, Tables S1–S3). The results showed an increase in *K*_m_^app^ for 2OG in the presence of inhibitor [from 3 μM (no inhibitor) to 44 μM at 300 μM succinate, and to 18 μM at 100 μM oxaloacetate], while the *V*_max_ values remained the same, revealing that both succinate and oxaloacetate inhibit KDM5B in a 2OG competitive manner. The determined *K*_i_ values were 27 ± 6 μM for succinate and 54 ± 6 μM for oxaloacetate.

**Table 2:** Kinetic parameters for KDM5B

	*K*_m_(2OG)^app^ (μM)	*K*_m_(H3K4me_2_(1–21))^app^ (μM)	*k*_cat_ (s^− 1^)	*K*_*i*_ (succinate; μM	*K*_*i*_ (oxaloacetate; μM)
KDM5B	2.6 ± 0.6	~ 0.5	0.015 ± 0.01	27 ± 6	54 ± 6

Assay conditions: 0.6 μM KDM5B. Variable concentrations of 2OG and H3K4me_2_(1–21) peptide: 10 μM Fe(II), 500 μM L-ascorbate in 50 mM Hepes, 50 mM NaCl (pH 7.5). Errors represent 95% confidence intervals. The levels of methylated and demethylated peptides were analyzed using MALDI-TOF-MS.

### Co-crystal structures of complexes of KDM5B with TCA cycle metabolites provide evidence for 2OG competitive binding

**Fig. 2:**
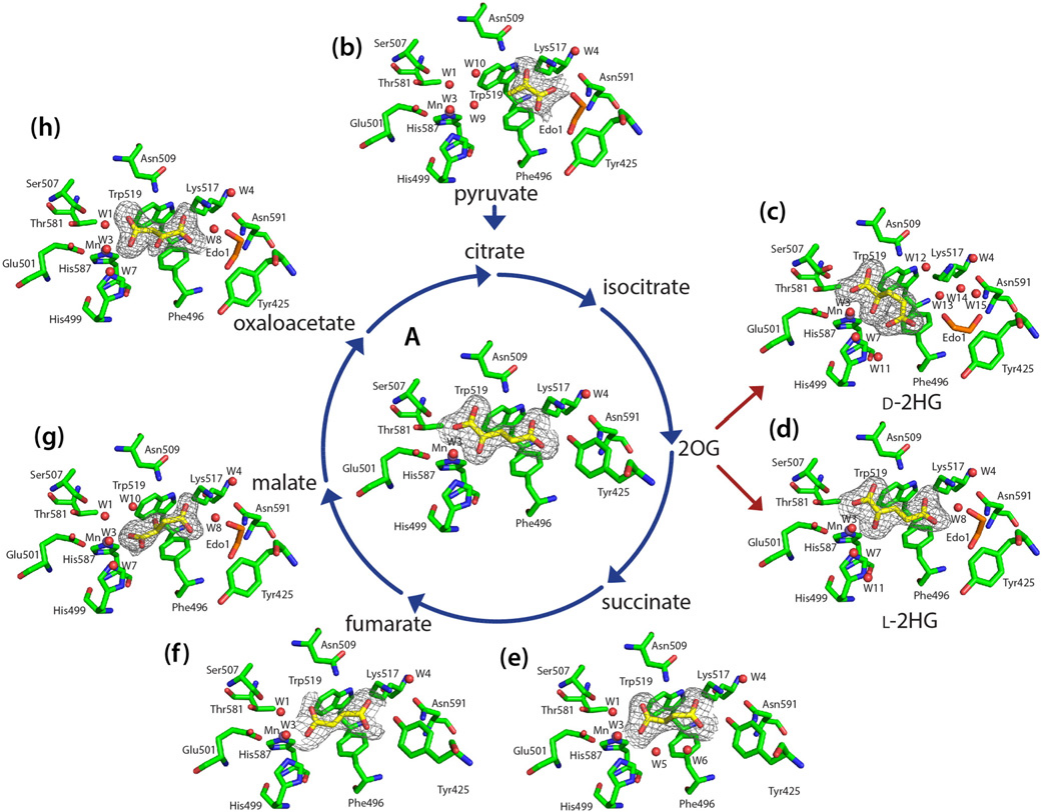
Views from crystal structures of KDM5B in complex with TCA cycle intermediates and the oncometabolites L- and D-2HG. Residues in the active site of KDM5B are shown as green sticks, whereas the TCA intermediates (a–b, e–h) and L- and D-HG (c and d) are shown as yellow sticks. Mn(II) (substituting for Fe(II)) is shown as an purple sphere, and water molecules as red spheres. Ethylene glycol derived from the cryoprotectant is shown in orange sticks. The 2*F*_o_–*F*_c_ map for the ligands is contoured at 1*σ*. The arrows reflect the TCA cycle and associated reactions (e.g., D-2HG levels are elevated due to mutations to DNA encoding for isocitrate dehydrogenases).

To investigate the mode of binding of the TCA cycle metabolites to KDM5B, we utilized recombinant KDM5B (F26-K772) lacking its ARID and PHD1 domain, which we have previously successfully used in crystallization studies [8]. Crystals of the KDM5B·Mn(II) complex were soaked with either 2OG, succinate, fumarate, malate, oxaloacetate, pyruvate, D- and L-2HG, citrate, or isocitrate. Ligand structure complexes were obtained for all intermediates (resolutions 1.8–2.5 Å), except for citrate and isocitrate (Fig. 2), consistent with the observed weak binding and absence of inhibition for these compounds.

It has been previously reported that 2OG is bound at the active site of KDM5B in a manner typical for other 2OG oxygenases, including the JmjC subfamily [8], [47], [48]; 2OG coordinates the active site metal in a bidentate manner via one of its C-1 carboxylate and C-2 keto oxygens. Octahedral metal coordination is completed by His587, His499 and Glu501, and a water molecule (W3, Fig. 2a). According to the consensus mechanism for 2OG oxygenases, binding of the metal bound water is weakened on substrate binding, hence promoting the formation of a vacant coordination site for dioxygen to bind [10]. In addition to binding the metal, the Glu501 side-chain carboxylate may be involved in interactions with, and the positioning of, the *N*^*ε*^-methyl lysine group of the substrate. The C-1 carboxylate of 2OG is further stabilized by hydrogen bonding with Ser507, which may be involved in 2OG decarboxylation/loss of CO_2_ during catalysis [8]. The C-5 carboxylate of 2OG is positioned to form electrostatic interactions with Lys517 and Asn509, via one of its oxygens, whereas the other C-5 carboxylate oxygen forms a hydrogen bonding interaction with the hydroxyl of Tyr425. The side chains of Trp514 and Phe496 are positioned to clamp the C-3 and C-4 methylenes of 2OG via hydrophobic interactions.

The studied TCA cycle intermediates all bind in the 2OG pocket (Fig. 2b–h). All, except for D-2HG, are positioned to form electrostatic interactions with Lys517 in an approximately similar position to that of the C-5 2OG carboxylate. In the case of succinate and fumarate, the C-5 2OG carboxylate equivalent is positioned to form additional interactions with the hydroxyl group of Tyr425 (Fig. 2e, f). For all the other ligands, the Tyr425 side chain is directed away from the active site (Fig. 2b–d, g–h). Asn509, which has previously been observed to adopt more than one conformation [8], is involved in the formation of electrostatic and hydrogen bond interactions with carboxylates of the ligands (Fig. 2). In the succinate complex, Asn509 is located as observed for 2OG and is positioned to form interactions with both succinate carboxylates (Fig. 2e). In the other complex structures, except that for pyruvate, the Asn509 side chain is positioned to form interactions between its primary amide and the ligand carboxylate equivalent to the 2OG C-2 carboxylate (2.9–3.5 Å). In the pyruvate complex, the Asn509 amide nitrogen is instead positioned to form electrostatic interactions (distance 3.1 Å) with C-2 ketone carbonyl oxygen.

Interestingly, in the case of oxaloacetate, the ligand binds such that its C-4 carboxylate, rather than its 2-oxoacid (as observed with 2OG), ligates to the metal. This binding mode should enable discrimination between oxaloacetate and 2OG with respect to oxidative decarboxylation. It may reflect a form of negative catalytic regulation; that is, oxaloacetate binds in a catalytically non-productive manner to avoid oxidative decarboxylation. Like oxaloacetate, pyruvate binds with its carboxylate occupying the 2OG C-5 carboxylate equivalent position; however, the ketone oxygens of oxaloacetate and pyruvate project in opposite directions.

The binding of L-2HG is similar to that of 2OG, with the C-2 hydroxyl group occupying a similar position to the C-2 carbonyl of 2OG and the C5 carboxylate is found in an identical position. However, in the case of D-2HG, the C-5 carboxylate is rotated out of the binding pocket to form interactions with water molecules (W3, W7 and W11), while its C-1 carboxylate and C-2 OH-group bind similarly as in 2OG (Fig. 2c).

**Fig. 3:**
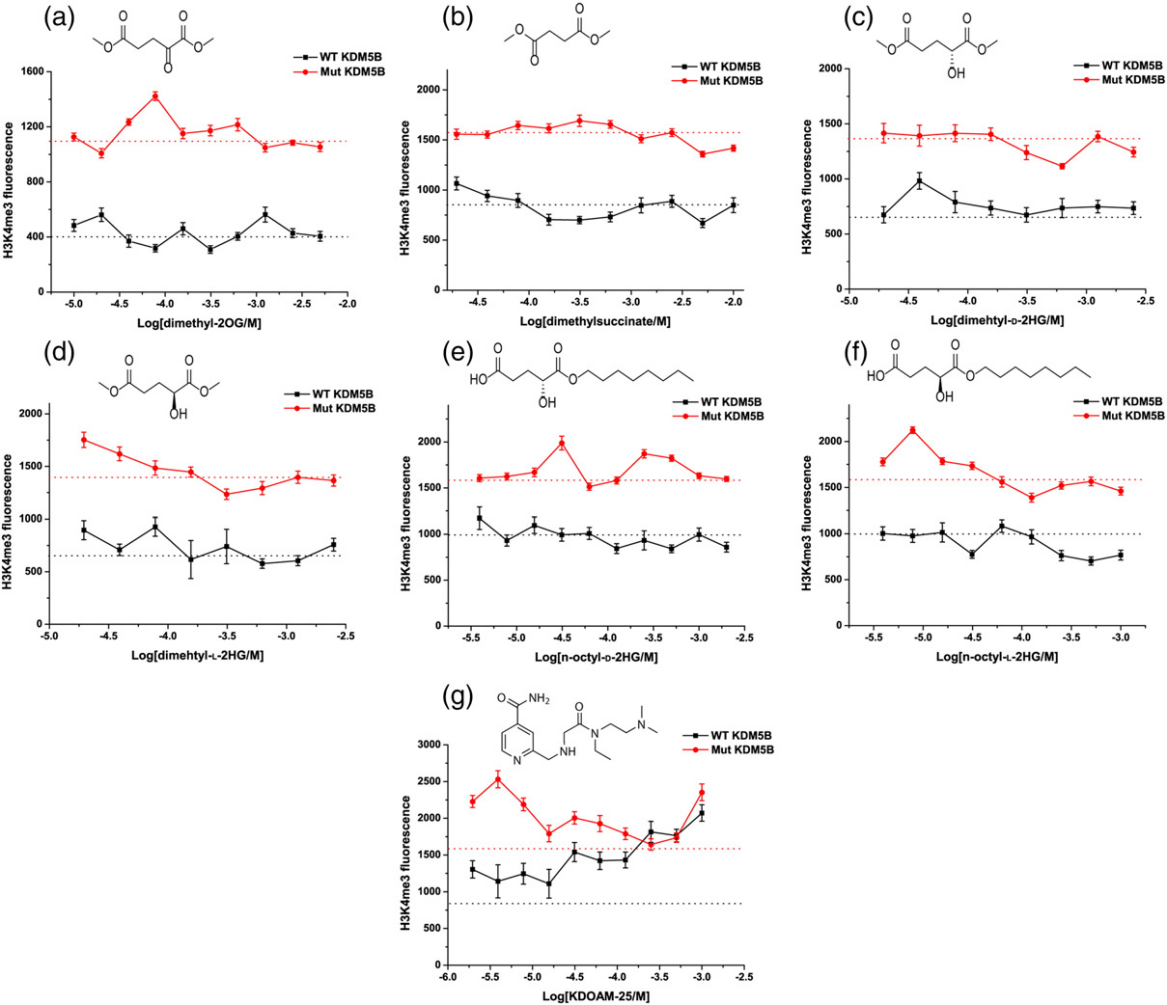
TCA cycle-related metabolites (administered as pro-drug esters) do not inhibit the activity of over-expressed KDM5B in U2-OS cells. EC_50_ curves for cells overexpressing Flag-tagged KDM5B. IF assay showing the effects of WT KDM5B-mediated H3K4me_3_ demethylation in comparison to its inactive mutant (Mut KDM5B). Results for dimethyl-2OG (a), dimethyl-succinate (b), dimethyl-D-2HG (c), dimethyl-L-2HG (d), octyl-D-2HG (e), octyl-L-2HG (f), and KDOAM-25 (g). Dashed lines represent IF levels in non-inhibitor controls.

Different solvation patterns relative to 2OG were observed for the studied ligands, with additional water and, sometimes, ethylene glycol molecules present within the 2OG binding pocket (Fig. 2), likely reflecting the weak binding of the relevant ligands. Additional water molecules were sometimes observed/refined in the metal or C-1 2OG carboxylate binding region, in some cases likely due to the smaller size of the bound ligand relative to 2OG (e.g., pyruvate; Fig. 2b); in other cases, they may reflect additional/altered hydrogen bonding opportunities enabled by the ligand structure (e.g., L-2HG/D-2HG; Fig. 3c, d). The β5–β6 loop, which contains the JmjN–ARID linkage, is found to be ordered only in the crystal structure in complex with pyruvate and is positioned as reported [8].

### Studies of KDM5B inhibition by TCA cycle metabolites in cells do not show elevated levels of H3K4me_3_

The cellular effects of dimethyl ester precursors of TCA cycle-related metabolites as potential inhibitors of H3K4me_3_ demethylation were then assessed. We used an established immunofluorescence (IF) procedure with ectopically overexpressed wild-type KDM5B (WT KDM5B) in U2-OS cells and anti-H3K4me_3_ antibody monitoring for changes in H3K4me_3_ methylation levels [49]. The demethylation activity of WT KDM5B in cells was compared to that of its catalytically inactive variant (H499A/E501A, hereafter, KDM5B Mut) [50]. Cells with overexpressed KDM5B were treated with different concentrations of the TCA cycle-related pro-drug esters (previously established as cell permeable in other cell-based studies) [28], [51], [52], [53], [54] for 24 h, the H3K4me_3_ levels were then analyzed in the transfected cells. The reported KDM5B inhibitor KDOAM-25 ((2)-((((2)-(((2)-(dimethylamino)ethyl)(ethyl)amino)-(2)-oxoethyl)amino)methyl)isonicotinamide) was used as a positive control for inhibition [50], [55]. Comparison of cells transfected with WT KDM5B or KDM5B Mut expressing vectors showed the expected decrease in H3K4me_3_ levels in the WT transfected cells (Fig. 3).

We first investigated potential changes in H3K4me_3_ levels in response to different concentrations of dimethyl-2OG (Fig. 3a). No decrease in the H3K4me_3_ levels was observed, suggesting that 2OG levels are saturating for KDM5B activity in the tested cells. Although other factors may be involved in cells, this observation is consistent with the relatively low *K*_m_^app^(2OG) (3 μM) for KDM5B determined with isolated recombinant KDM5B. The esters of the TCA cycle metabolites (dimethyl-2OG, dimethylfumarate, dimethyl-D-/dimethyl-L-2HG, octyl-D-/octyl-L-2HG) were thus dosed up to 5–10 mM concentration. In contrast with the experiments with isolated KDM5B, no inhibition of KDM5B activity was observed (Fig. 3b). Dimethylfumarate was toxic at concentrations higher than 400 μM and did not show any inhibition at lower concentrations (data not shown). Inhibition by oxaloacetate in cells was not studied due to its unstable nature [56]. By contrast, KDOAM-25 (a potent positive control inhibitor, IC_50_ value is 19 nM against isolated KDM5B as determined by AlphaScreen assay [55]) manifested the expected levels of inhibition, with H3K4me_3_ fluorescence reaching levels of KDM5B Mut at compound concentration of 1 mM (Fig. 3g). Thus, 2OG competitive inhibition (as observed by kinetics and crystallography) may mitigate the inhibitory effects of the elevated levels of the studied TCA cycle-related compounds in cells.

## Discussion

Although the inhibition of 2OG oxygenases involved in chromatin regulation by TCA cycle intermediates and D-/L-2HG has been widely considered as of relevance in tumor biology, there are few systematic biochemical studies. Our results on the interaction of TCA cycle-related metabolites with isolated KDM5B reveal the potential for its inhibition by some, but not all, of the tested compounds, including D-2HG, levels of which are elevated in cancer cells due to mutations to IDH1/2 [46].

Crystal structures of KDM5B complexed with manganese (substituting for iron) and the TCA cycle-related metabolites reveal that most of the compounds have the potential to inhibit KDM5B, and by implication other KDM5 subfamily enzymes, in a 2OG competitive manner. Although, all of the inhibiting metabolites occupy the 2OG binding site, they exhibit variations in their binding modes, which appears to correlate with the observed potencies. Thus, pyruvate was shown to be a very weak KDM5B inhibitor and did not manifest metal chelation in its binding mode. Citrate and isocitrate were also inactive as KDM5B inhibitors under the studied assay conditions, consistent with our inability to obtain crystal structures for them.

The crystal structures provide evidence that the conformations of the 2OG binding pocket in KDM5B are relatively flexible (as manifested by the observed alternative conformations for Tyr425 and Asn509 [8]) compared to other JmjC oxygenases (e.g., the KDM4 subfamily D-/L-2HG, PDB ID: 2YBK and 2YBS, respectively). This property apparently enables the 2OG binding pocket in KDM5B to adapt to the different shapes of the various TCA cycle-related intermediates.

**Fig. 4:**
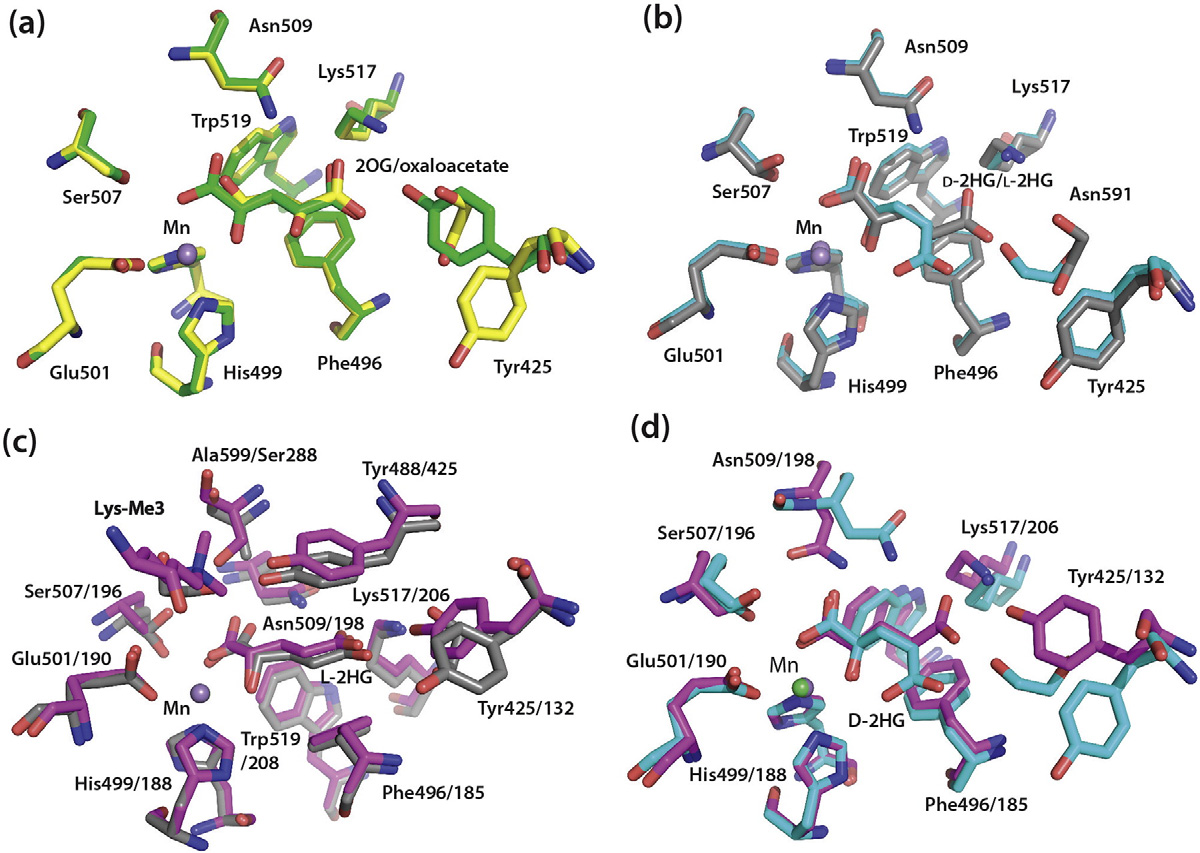
Comparison of the binding modes of TCA intermediates and L- and D-2HG within the 2OG binding pockets of KDM5B and KDM4A. (a) Overlay of a structure of KDM5B complexed with 2OG (green) or oxaloactetate (yellow). Tyr425 is observed in different conformations, one of which is replaced by solvent molecules in the structure with oxaloacetate. (b) Overlay of KDM5B complexed with L-2HG (gray) and D-2HG (cyan) showing alternative positions for the C-5 carboxylate. (c–d) Overlay of L-2HG (c) and D-2HG (d) complexed with KDM4A (magenta) and KDM5B (D-2HG, cyan; L-2HG, gray). Note the nearly identical binding modes for the L-2HG with KDM5B and KDM4A, but the different binding modes for the C-5 carboxylate of D-2HG.

The conformations of the 2OG binding pocket residues in complexes of D- and L-2HG with KDM4A [28] are similar to those observed for KDM5B (notably KDM4A-Ser288/KDM5B-Ala599, Fig. 4c, d); L-2HG binds in the same conformation to both enzymes; however, the C-5 carboxylate of D-2HG in KDM5B points toward the metal, forming water-mediated interactions, rather than the electrostatic interaction with conserved KDM4A-Lys206 (Fig. 4c, d). Notably, the trimethylated lysine in the H3K36me_3_(30–41) present in a L-2HG complex with KDM4A (PDB ID: 2YBS), superimposes with an ethylene glycol molecule present in all the KDM5B complexes, suggesting that the ethylene glycol occupies part of the position of the peptide substrate in the KDM5B active site.

Of the tested metabolites, succinate and oxaloacetate showed the highest potency for KDM5B inhibition, with IC_50_ values of 15 ± 10 and 62 ± 19 μM, respectively. While oxaloacetate has not been reported to accumulate under pathological conditions, increased succinate levels are established in certain cancers [19]. Comparison of the D-/L-2HG inhibition potencies for different 2OG oxygenases (Table 3) suggests that they differently affect human members of the 2OG oxygenase superfamily [as may also be the case for other (combinations of) TCA cycle-related metabolites]. Notably, D-2HG and L-2HG showed lower levels of KDM5B inhibition compared to that reported for KDM4A (obtained IC_50_ values of 203 ± 90 μ and 150 ± 40 μM for D-/L-2HG, respectively, compared to 25 μM for KDM4A; Table 3), consistent with our cellular results [28]. However, D-2HG levels can reach mM concentrations in cells, as seen in IDH1/2 mutations; thus, metabolite-specific inhibition of KDM5B (and by implication other KDM5 members) by D-2HG in tumors cannot be ruled out. It may also become relevant under cellular conditions when 2OG concentrations are reduced, such as in tumor cells where mutations to the TCA cycle enzymes disrupt the TCA cycle metabolite ratios [39], [46], [59].

**Table 3:** Reported results for inhibition of 2OG oxygenases by TCA cycle intermediates

Enzyme	Enzyme concentration (μM)	IC_50_ (μM)				*K*_m_^app^(2OG; μM)
		Succinate	Fumarate	D-2HG	L-2HG	
KDM4A	2 [28]	NR	NR	24 ± 2 [28]	26 ± 3 [28]	6 [28]
KDM4C	1 [28]	NR	NR	79 ± 7 [28]	97 ± 24 [28]	4 [28]
KDM4E	2 [57]	320 [57]	2.3 [57]	NR	NR	13.2 [58]
KDM2A	2 [28]	NR	NR	106 ± 22 [28]	48 ± 15 [28]	12.5 [28]
PHD2	0.5 [28]	19 [29]	3 [29]	7300 ± 3300 [28]	419 ± 150 [28]	13 ± 2 [45]
FIH	1 [28]	> 1000 [29]	> 1000 [29]	1500 ± 400 [28]	189 ± 34 [28]	110 ± 20 [45]
BBOX	0.025 [28]	NR	NR	13,200 ± 1100 [28]	142 ± 30 [28]	470 [45]
ABH2	2 [28]			424 ± 77 [28]	150 ± 20 [28]	10 [28]
TET1	NR	540 ± 100 [22]	390 ± 160 [22]	4000 [26]	1000 [26]	55 ± 20 [22]
TET2	NR	570 ± 190 [22]	400 ± 70 [22]	5000 [26]	1600 [26]	60 ± 15 [22]
KDM5B	0.6	62 ± 19	210 ± 80	203 ± 90	150 ± 40	2.6 ± 0.6

NR, not reported. Note that the assays were carried out under different conditions, including with respect to enzyme concentration.

In contrast with the results for some other 2OG oxygenases (e.g., the KDM4 demethylases and the TET enzymes) [22], [28], [39], [53], our cellular results (with over-expressed KDM5B) imply that KDM5B is not, at least strongly, regulated by changes in levels of the tested metabolites. We did, however, observe inhibition with the potent KDM5B inhibitor KDOAM-25. Changes in the intracellular levels of 2OG are proposed to influence 2OG oxygenase activity [38]; there are also considerable variations in the *K*_m_^app^ values for 2OG reported for 2OG oxygenases, which likely also influence their 2OG competitive inhibition. However, treatment of U2-OS cells with increasing amounts of dimethyl-2OG did not show any effect on the activity of ectopically overexpressed KDM5B to demethylate H3K4me_3_, suggesting that under the tested conditions, the intracellular 2OG levels are saturating for KDM5B catalysis. This result is consistent with the relatively low *K*_m_^app^(2OG) for KDM5B (3 μM), which is likely below intracellular 2OG concentrations [60].

Thus, although there are caveats on the physiological relevance of our cellular work with KDM5B (as with that for other JmjC KDMs), the available evidence is that some JmjC KDMs and, by implication histone marks, may be more susceptible to changes in specific metabolite concentrations than others (e.g., D-2HG, succinate or fumarate as can occur in tumors). The overall results, however, raise the possibility of regulation of KDM5 activity by competition for 2OG binding by multiple metabolites, the combined concentration of which will be high relative to 2OG. Such competition by multiple molecules has the potential to tune enzyme activity and to provide effects enabled by context- and time-dependent changes in the competing metabolic composition [61]. Such competition has been proposed as a regulatory mechanism for catalysis by the JmjC “hydroxylase” factor inhibiting hypoxia-inducible factor (FIH) [61]. However, in the case of FIH, the proposed “multiple-molecule” competition occurs between one substrate class “HIF-α isoforms” on which FIH catalysis has a profound signaling effect and another substrate class “ankyrins” on which FIH does not have an (identified) signaling role. Competition between HIF and ankyrins is proposed to regulate hydroxylation of the former [61].

Finally, it is important to note that many 2OG oxygenase inhibitors, including inhibitors in clinical development and KDM5 subfamily inhibitors (Supplementary Information, Fig. S3) [8], [55], bind in the 2OG binding pocket [23]. The combined results described here and previously suggest that it is possible that both the absolute and relative concentrations of TCA cycle-related intermediates could influence pharmaceutical inhibitor potency in vivo.

## Materials and Methods

### Recombinant KDM5B production and purification

Recombinant KDM5B (M1-R822 and F26-K722) was produced in Sf9 insect cells and purified as previously described [8].

### Crystallization and crystal structure determinations

A JmjN–JmjC linked construct of KDM5B (F26-K772) was crystallized using the sitting drop vapor diffusion method as previously described [8]. Crystals of KDM5B were soaked with final concentrations of 10–25 mM of the TCA intermediates supplemented with 25% (v/v) ethylene glycol for 15 to 120 min and then flash frozen in liquid nitrogen. Data were collected at Diamond Light Source beamlines I04-1, I02 and I04. Data sets were processed using Xia2 [62], molecular replacement and initial refinement in DIMPLE pipeline [63] (used the PDB ID: 5A1F as a model). Iterative model building was done using COOT [64] and refinement with PHENIX [65] and BUSTER v2.10.1 [66]. Data collection and refinement statistics are given in Table S3, Supplementary Information.

### MALDI-TOF-MS-based enzymatic assays

Kinetic assays were performed in 50 mM Hepes, 50 mM NaCl (pH 7.5) buffer at 25 °C. KDM5B (0.6 μM) was incubated with various amounts of 2OG and H3K4me_2_(1–21) peptide (a fragment of the natural histone/nucleasome substrate), 10 μM Fe(NH_4_)_2_(SO_4_)_2_ and 500 μM sodium L-ascorbate. The reaction was quenched with 1% formic acid at defined time points. The samples were then mixed in 1:1 ratio with α-cyano-4-hydroxycinnamic acid matrix and spotted onto a MALDI target plate. Spectra were acquired using MALDI-TOF-MS (Micro MX, Waters, UK) in reflectron positive mode and processed using MASSLYNX4.0. Time courses at each 2OG/peptide concentrations were analyzed and initial rates of demethylation were determined. The initial rates were plotted *versus* concentration and analyzed with the assumption of Michaelis–Menten kinetics using Origin 8.51 software.

Inhibition assays were performed in 50 mM Hepes, 50 mM NaCl (pH 7.5) buffer at 25 °C. KDM5B (0.6 μM) was incubated with 3 μM disodium 2OG, 5 μM H3K4me_2_(1–21), 10 μM Fe(NH_4_)_2_(SO_4_)_2_ and 500 μM sodium L-ascorbate for 5 min and subsequently quenched with 1% formic acid. The samples were mixed in a 1:1 ratio with α-cyano-4-hydroxycinnamic acid matrix and spotted onto a MALDI target plate for MS analysis. The demethylation levels were normalized compared to the non-inhibitor control. The IC_50_ values were determined using GraphPad Prism software. All experiments were carried out in triplicate.

### Cellular assays

Immunofluorescent cell-based assays were performed as reported [50]. The human U2-OS osteosarcoma cells were cultured as a monolayer in Dulbecco's modified Eagle's medium (Invitrogen) supplemented with 10% heat-inactivated fetal bovine serum (Life Technologies) and 1% Glutamax. Full-length cDNA encoding for WT KDM5B or the catalytically inactive variant KDM5B H499A/E501A, both with a 3*FLAG N-terminal tag, was inserted into the pCDNA5-FRT-TO-3FLAG vector to ectopically overexpress the genes [50]. Cells were transiently transfected using cDNA and Lipofectamine 3000 (Life Technologies). Four hours after transfection, the cells were subjected to treatment with serial dilutions of compound. After 24 h, the cells were fixed with 4% formaldehyde and stained with anti-Flag antibody (Sigma F3165), an anti-methylated histone antibody (H3K4me_3_ DiagenodeC15410003). The cells were then stained with secondary antibodies [Alexa Fluor 488 and Alexa Fluor 568-labeled (Life Technologies)] and 4’,6-diamidino-2-phenylindole. Image acquisition was conducted using Operetta High Content Imaging System (PerkinElmer), and image analysis was performed using the Columbus software (PerkinElmer). A minimum threshold for anti-Flag intensity was set to ensure that only cells highly producing the demethylase were analyzed.

### Synthesis of esters of TCA cycle intermediates and KDOAM-25

KDOAM-25 was kindly provided by Dr. Gian-Filippo Ruda and Prof. Paul Brennan (Target Discovery Institute, Structural Genomics Consortium, University of Oxford) [55]. Dimethyl-L-2HG and dimethyl-D-2HG were synthesized according to the described procedures [28], [55]. Dimethyl-2-oxoglutarate, dimethylsuccinate and dimethylfumarate were purchased from Sigma-Aldrich, and octyl-L-2HG and octyl-D-2HG were purchased from Cambridge Bioscience.

### PDB accession numbers

5FUP (2OG), 5FY4 (succinate), 5FY5 (fumarate), 5FYV (oxaloacetate), 5FY9 (pyruvate), 5FZ8 (malate), 5FYS (D-2-hydroxygutarate), 5FZD (L-2-hydroxyglutarate).

## Appendix A Supplementary data

Supplementary material Image 2
