# Supplementary material for: Studies on the Interaction of the Histone Demethylase KDM5B with Tricarboxylic Acid Cycle Intermediates
Source: J Mol Biol. 2017 Sep 15;429(19):2895–906. doi: 10.1016/j.jmb.2017.08.007 (PMC5636616; doi:10.1016/j.jmb.2017.08.007)
Supplement: Supplementary file 1 — Supplementary material [file mmc1.docx]

**Supplementary Information**

**Studies on the Interaction of the Histone Demethylase KDM5B with Tricarboxylic Acid Cycle Intermediates**

Hanna Tarhonskaya^ǂ1^, Radoslaw P. Nowak^ǂ2^, Catrine Johansson^1,3^, Aleksandra Szykowska^2^, Anthony Tumber^2^, Rebecca L. Hancock^1^, Pauline Lang^1^, Emily Flashman^1^, Udo Oppermann^2^, Christopher J. Schofield^1*^ and Akane Kawamura^1*^

*^1^Chemistry Research Laboratory, University of Oxford, Department of Chemistry, 12 Mansfield Road, Oxford, OX1 3TA, United Kingdom.*

*^2^Structural Genomic Consortium, University of Oxford, Old Road Campus, Roosevelt Drive, Oxford, OX3 7DQ, United Kingdom.*

*^3^Botnar Research Centre, NIHR Oxford Biomedical Research Unit, University of Oxford, Windmill Road, Oxford, OX3 7LD, United Kingdom.*

^ǂ^These authors contributed equally to this work.

*To whom correspondence should be addressed: [christopher.schofield@chem.ox.ac.uk](mailto:christopher.schofield@chem.ox.ac.uk), [akane.kawamura@chem.ox.ac.uk](mailto:akane.kawamura@chem.ox.ac.uk)


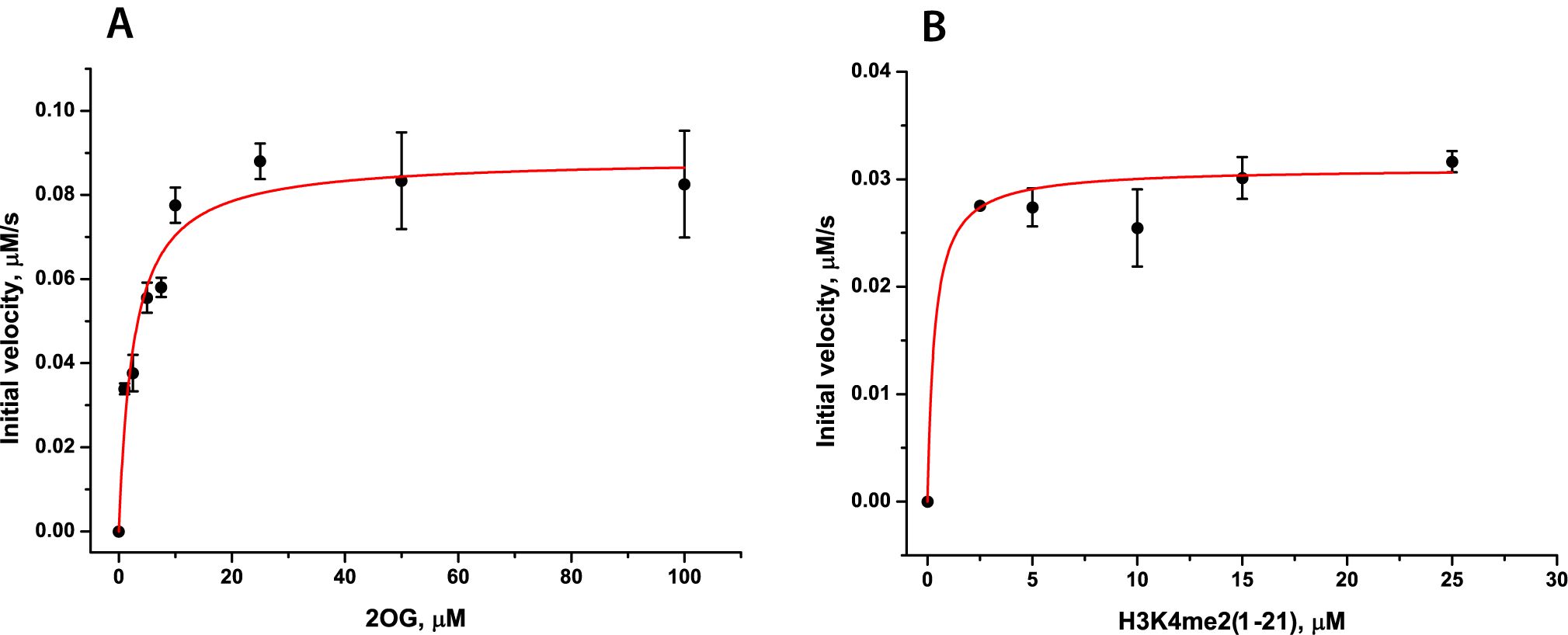


**Figure S1. Determination of kinetic parameters for KDM5B with H3K4me_2_(1-21). A.** Conditions: 1 µM KDM5B was incubated with different concentrations of 2OG, 5 µM H3K4me_2_(1-21), 10 µM Fe(II), 500 µM L-ascorbate in 50 mM HEPES 150 mM NaCl (pH 7.5). **B.** Conditions: 0.6 µM KDM5B was incubated with 10 µM 2OG, various concentrations of H3K4me_2_(1-21), 10 µM Fe(II), 500 µM L-ascorbate in 50 mM HEPES 150 mM NaCl pH 7.5. Error bars represent standard deviations for triplicate assays. The levels of methylated and demethylated peptides were analysed by MALDI-TOF-MS.


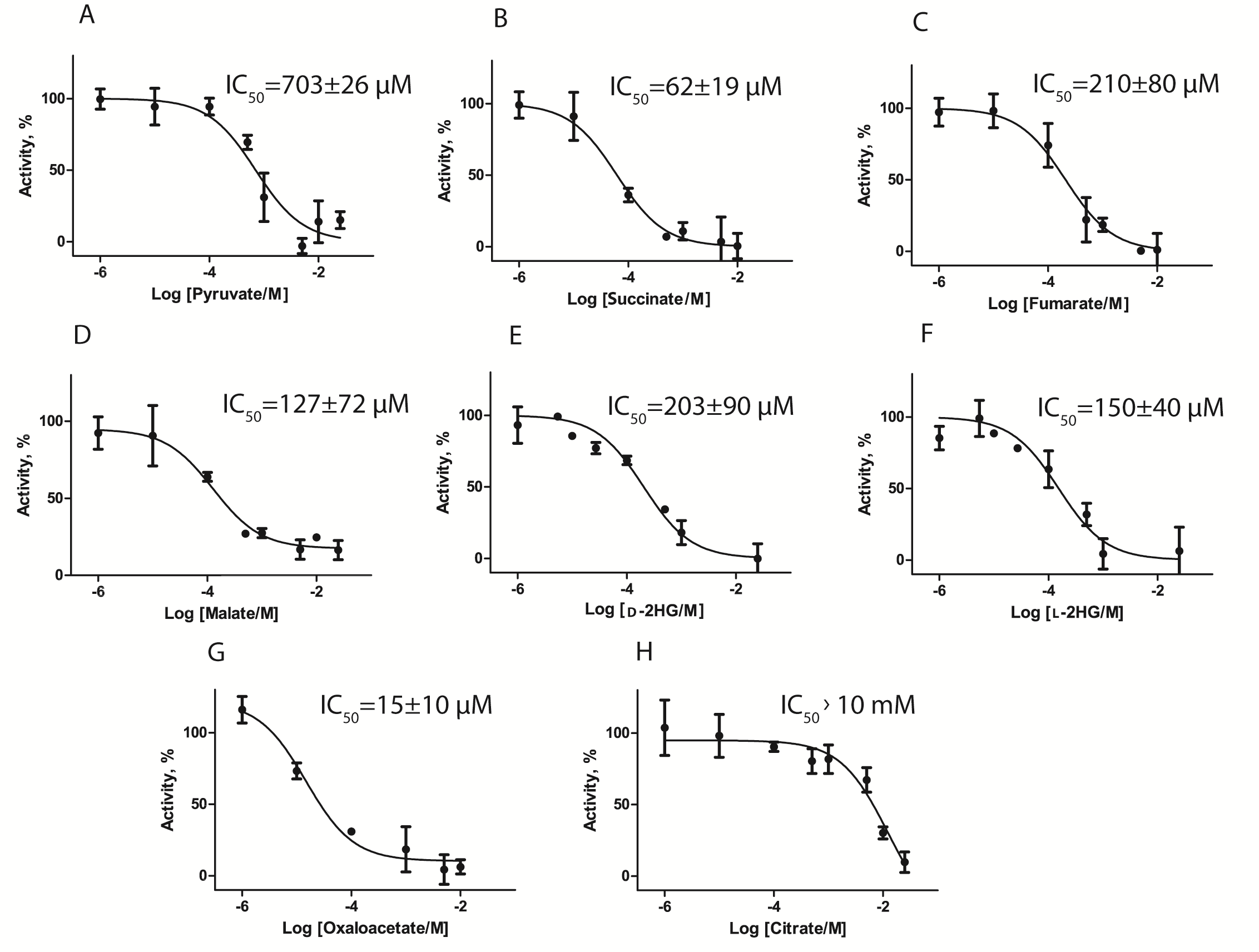


**Figure S2. Inhibition of KDM5B by TCA cycle intermediates. A.** pyruvate, **B.** succinate, **C.** fumarate, **D.** malate, **E.** D-2HG, **F.** L-2HG, **G.** oxaloacetate, **H.** citrate. Assay conditions: 0.6 µM KDM5B was incubated with 3 µM 2OG, 5 µM H3K4me_2_(1-21), 10 µM Fe(II), 500 µM L-ascorbate in 50 mM HEPES 50 mM NaCl (pH 7.5). Error bars represent standard deviations for triplicate assays. The levels of methylated and demethylated peptides were analysed by MALDI-TOF-MS.

**
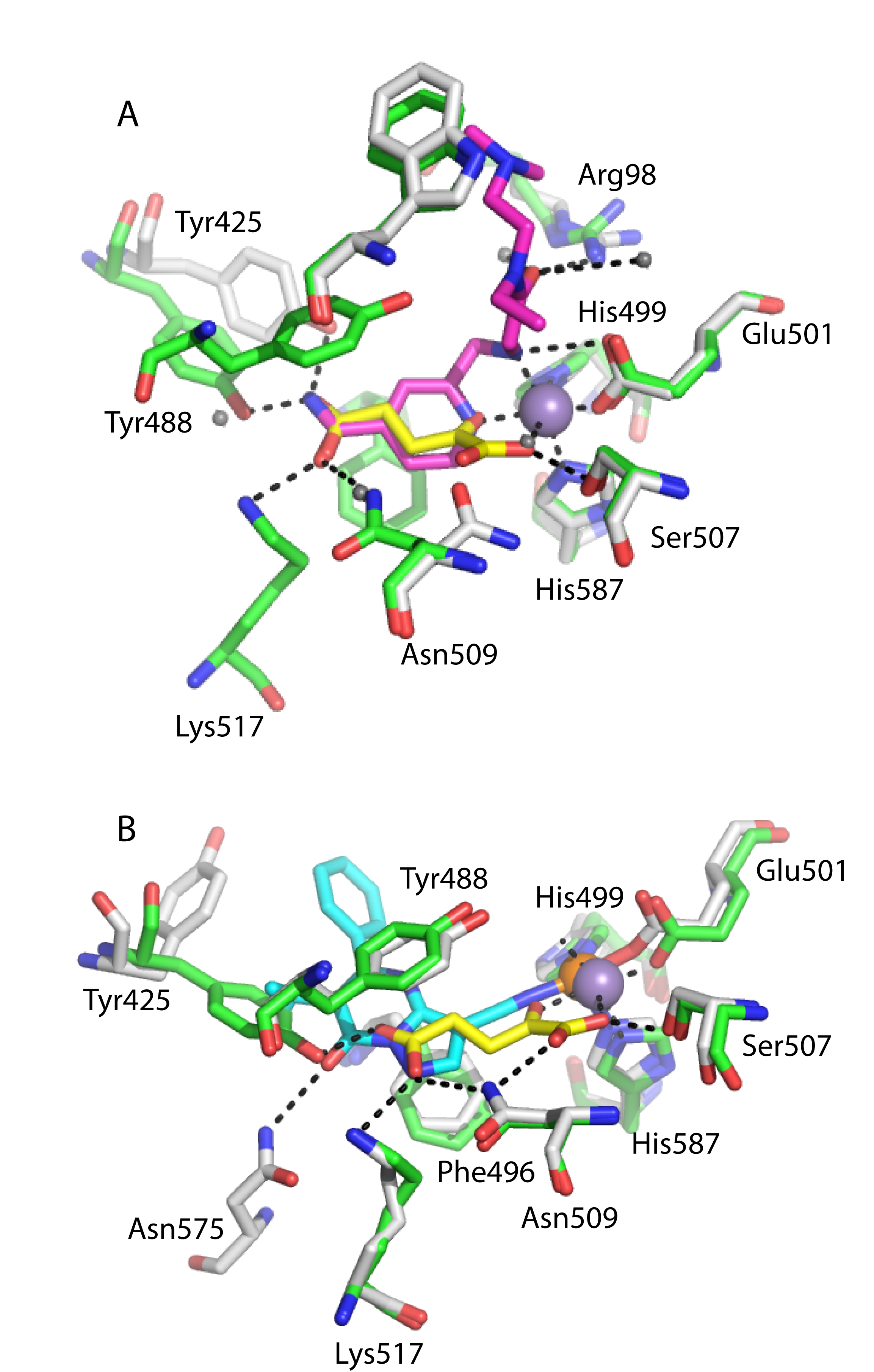
**

**Figure S3: Views from the crystal structures of KDM5B in complex with inhibitors occupying the 2OG binding pocket.** Active site of KDM5B (green) in complex with 2OG (yellow) (PDB ID 5FUP) overlaid with (A) KDM5B:KDOAM25A complex (PDB ID 5A3N, silver and magenta respectively) and (B) KDM5A:CPI-455 complex (PDB ID 5A3N, silver and cyan respectively). Metal ions are shown as purple and orange spheres, and water molecules in the KDM5B:KDOAM25 complex as grey spheres. Amino acid residues are labelled as in PDB ID 5FUP. Note the overlap of the inhibitor and 2OG binding sites.

## .Table S1. Results of K_i_ determination for succinate using GraphPad Prism 5.0.

## The data were globally fitted using a competitive inhibition model.

| **Competitive inhibition** |  |  |  |  |  |
| --- | --- | --- | --- | --- | --- |
| **Best-fit values** |  |  |  |  |  |
| **Km** | 3.006 | 3.006 | 3.006 | 3.006 | 3.006 |
| **I** | **= 0.0** | **= 30.00** | **= 100.0** | **= 300.0** |  |
| **Ki** | 26.77 | 26.77 | 26.77 | 26.77 | 26.77 |
| **Vmax** | 0.004450 | 0.004450 | 0.004450 | 0.004450 | 0.004450 |
| **Std. Error** |  |  |  |  |  |
| **K_m_** | 0.5003 | 0.5003 | 0.5003 | 0.5003 | 0.5003 |
| **K_i_** | 5.583 | 5.583 | 5.583 | 5.583 | 5.583 |
| **V_max_** | 0.0001377 | 0.0001377 | 0.0001377 | 0.0001377 | 0.0001377 |
| **95% Confidence Intervals** |  |  |  |  |  |
| **K_m_** | 1.989 to 4.023 | 1.989 to 4.023 | 1.989 to 4.023 | 1.989 to 4.023 | 1.989 to 4.023 |
| **K_i_** | 15.42 to 38.13 | 15.42 to 38.13 | 15.42 to 38.13 | 15.42 to 38.13 | 15.42 to 38.13 |
| **V_max_** | 0.004170 to 0.004730 | 0.004170 to 0.004730 | 0.004170 to 0.004730 | 0.004170 to 0.004730 | 0.004170 to 0.004730 |
| **Goodness of Fit** |  |  |  |  |  |
| **Degrees of Freedom** |  |  |  |  | 34 |
| **R square** | 0.9523 | 0.9085 | 0.9725 | 0.9713 | 0.9588 |
| **Absolute Sum of Squares** | 7.451e-007 | 1.560e-006 | 5.337e-007 | 5.004e-007 | 3.339e-006 |
| **Sy.x** |  |  |  |  | 0.0003134 |
| **Constraints** |  |  |  |  |  |
| **K_m_** | K_m_ > 0.0 and shared | K_m_ > 0.0 and shared | K_m_ > 0.0 and shared | K_m_ > 0.0 and shared |  |
| **I** | I = 0.0 | I = 30.00 | I = 100.0 | I = 300.0 |  |
| **K_i_** | K_i_ > 0.0 and shared | K_i_ > 0.0 and shared | K_i_ > 0.0 and shared | K_i_ > 0.0 and shared |  |
| **V_max_** | V_max_ > 0.0 and shared | V_max_ > 0.0 and shared | V_max_ > 0.0 and shared | V_max_ > 0.0 and shared |  |
| **Number of points** |  |  |  |  |  |
| **Analyzed** | 9 | 9 | 9 | 10 |  |

## Table S2. Results of K_i_ determination for oxaloacetate using GraphPad Prism 5.0. The data were globally fitted using a competitive inhibition model.

| **Competitive inhibition** |  |  |  |  |  |
| --- | --- | --- | --- | --- | --- |
| **Best-fit values** |  |  |  |  |  |
| **K_m_** | 5.905 | 5.905 | 5.905 | 5.905 | 5.905 |
| **I** | **= 0.0** | **= 15.00** | **= 50.00** | **= 100.0** |  |
| **K_i_** | 54.00 | 54.00 | 54.00 | 54.00 | 54.00 |
| **V_max_** | 0.006615 | 0.006615 | 0.006615 | 0.006615 | 0.006615 |
| **Std. Error** |  |  |  |  |  |
| **K_m_** | 0.5081 | 0.5081 | 0.5081 | 0.5081 | 0.5081 |
| **K_i_** | 6.109 | 6.109 | 6.109 | 6.109 | 6.109 |
| **V_max_** | 0.0002073 | 0.0002073 | 0.0002073 | 0.0002073 | 0.0002073 |
| **95% Confidence Intervals** |  |  |  |  |  |
| **K_m_** | 4.860 to 6.950 | 4.860 to 6.950 | 4.860 to 6.950 | 4.860 to 6.950 | 4.860 to 6.950 |
| **K_i_** | 41.44 to 66.56 | 41.44 to 66.56 | 41.44 to 66.56 | 41.44 to 66.56 | 41.44 to 66.56 |
| **V_max_** | 0.006189 to 0.007042 | 0.006189 to 0.007042 | 0.006189 to 0.007042 | 0.006189 to 0.007042 | 0.006189 to 0.007042 |
| **Goodness of Fit** |  |  |  |  |  |
| **Degrees of Freedom** |  |  |  |  | 26 |
| **R square** | 0.9869 | 0.9907 | 0.9797 | 0.9943 | 0.9889 |
| **Absolute Sum of Squares** | 2.669e-007 | 1.782e-007 | 3.110e-007 | 1.220e-007 | 8.780e-007 |
| **Sy.x** |  |  |  |  | 0.0001838 |
| **Constraints** |  |  |  |  |  |
| **K_m_** | K_m_ > 0.0 and shared | K_m_ > 0.0 and shared | K_m_ > 0.0 and shared | K_m_ > 0.0 and shared |  |
| **I** | I = 0.0 | I = 15.00 | I = 50.00 | I = 100.0 |  |
| **K_i_** | K_i_ > 0.0 and shared | K_i_ > 0.0 and shared | K_i_ > 0.0 and shared | K_i_ > 0.0 and shared |  |
| **V_max_** | V_max_ > 0.0 and shared | V_max_ > 0.0 and shared | V_max_ > 0.0 and shared | V_max_ > 0.0 and shared |  |
| **Number of points** |  |  |  |  |  |
| **Analyzed** | 7 | 7 | 7 | 8 |  |

**Table S3. Results of determination of 2OG K_m_^app^ in the presence of different concentrations of succinate and oxaloacetate.** The data were analysed by non-linear regression using GraphPad Prism 5.0.

| **Succinate, μM** | **V_m_^app^, μM/s** | **K_m_^app^, μM** | **Oxaloacetate, μM** | **V_m_^app^, μM/s** | **K_m_^app^, μM** |
| --- | --- | --- | --- | --- | --- |
| 0 | 0.0043 | 3.4 | 0 | 0.0062 | 5.0 |
| 30 | 0.0044 | 4.0 | 15 | 0.0066 | 7.2 |
| 100 | 0.0046 | 19.5 | 50 | 0.0070 | 15.0 |
| 300 | 0.0046 | 44 | 100 | 0.0069 | 18.5 |

**Table S4. Data collection and refinement statistics.**

|  | **2OG**  5FUP | **Succinate**  5FY4 | **Fumarate**  5FY5 | **Oxaloacetate**  5FYV | **Pyruvate**  5FY9 | **Malate**  5FZ8 | **D-2HG**  5FYS | **L-2HG**  5FZD |
| --- | --- | --- | --- | --- | --- | --- | --- | --- |
| **Wavelength (Å)** | 0.9173 | 0.9173 | 0.9173 | 0.9795 | 0.9795 | 0.9795 | 0.9173 | 0.9173 |
| **Resolution range (Å)** | 71.17  -2.15 (2.227  - 2.15) | 71.07 -2.1 (2.175  - 2.1) | 64.28 -2.47 (2.558  - 2.47) | 57.07  -1.87  (1.937  -1.87) | 57.13 -2.03  (2.103 -2.03) | 61.57 -1.86 (1.926 - 1.86) | 47.88 -1.89 (1.958 - 1.89) | 47.69  - 2.05 (2.123 - 2.05) |
| **Space group** | P 65 2 2 | P 65 2 2 | P 65 2 2 | P 65 2 2 | P 65 2 2 | P 65 2 2 | P 65 2 2 | P 65 2 2 |
| **Unit cell**  a(Å),b(Å),c(Å)  α β γ | 142.34 142.34 152.26 90 90 120 | 142.14 142.14 152.1  90 90  120 | 141.68 141.68 153  90 90  120 | 142.18 142.18 152.15  90 90  120 | 142.29 142.29 152.65 90 90 120 | 142.2 142.2 152.47 90 90 120 | 142.08 142.08 152.47 90 90 120 | 141.8 141.8 151.41 90 90 120 |
| **Total reflections** | 962030 (95849) | 1057061 (102944) | 623357 (64550) | 1488447 (144030) | 1173428 (114395) | 1516065 (144566) | 1438569 (143766) | 1108750 (112488) |
| **Unique reflections** | 49913 (4898) | 53306 (5232) | 33072 (3235) | 75058 (7363) | 59212 (5808) | 76431 (7507) | 72769 (7169) | 56673 (5560) |
| **Multiplicity** | 19.3 (19.6) | 19.8 (19.7) | 18.8 (20.0) | 19.8  (19.6) | 19.8 (19.7) | 19.8 (19.3) | 19.8 (20.1) | 19.6 (20.2) |
| **Completeness (%)** | 100.00 (100.00) | 100.00 (100.00) | 99.99 (100.00) | 99.99 (100.00) | 99.99 (100.00) | 99.99 (99.96) | 99.97 (99.80) | 100.00 (100.00) |
| **Mean I/sigma(I)** | 29.51 (5.25) | 19.90 (1.50) | 10.31 (1.51) | 23.02  (1.35) | 18.29 (1.50) | 15.64 (1.22) | 18.85 (1.36) | 15.78 (1.30) |
| **Wilson B-factor** | 38.12 | 45.47 | 57.76 | 37.06 | 40.40 | 36.15 | 35.77 | 41.76 |
| **R merge † (%)** | 0.06963 (0.6652) | 0.1085 (2.232) | 0.2032 (2.196) | 0.08522 (2.097) | 0.1325 (2.432) | 0.1153 (2.334) | 0.1097 (2.566) | 0.1434 (2.398) |
| **R-meas** | 0.07154 | 0.1114 | 0.209 | 0.08747 | 0.136 | 0.1184 | 0.1126 | 0.1472 |
| **CC1/2** | 1 (0.954) | 0.999 (0.571) | 0.994 (0.64) | 1  (0.563) | 0.999 (0.598) | 0.999 (0.533) | 1 (0.539) | 0.999 (0.624) |
| **CC*** | 1 (0.988) | 1  (0.853) | 0.999 (0.884) | 1  (0.849) | 1  (0.865) | 1  (0.834) | 1  (0.837) | 1  (0.877) |
| **R-work‡** | 0.1830 (0.2454) | 0.1977 (0.3096) | 0.1909 (0.2835) | 0.1991 (0.3230) | 0.2253 (0.3196) | 0.2061 (0.3341) | 0.1949 (0.3144) | 0.1970 (0.3175) |
| **R-free§** | 0.2259 (0.3105) | 0.2337 (0.3581) | 0.2263 (0.3297) | 0.2333 (0.3387) | 0.2559 (0.3701) | 0.2402 (0.3563) | 0.2261 (0.3392) | 0.2372 (0.3568) |
| **Number of non-hydrogen atoms** | 4263 | 3935 | 3855 | 4242 | 3904 | 4161 | 4266 | 4256 |
| **macromolecules** | 3708 | 3667 | 3727 | 3750 | 3762 | 3770 | 3768 | 3762 |
| **ligands** | 66 | 50 | 60 | 72 | 69 | 68 | 73 | 73 |
| **water** | 489 | 218 | 68 | 420 | 73 | 323 | 425 | 421 |
| **Protein residues** | 454 | 465 | 452 | 453 | 453 | 453 | 453 | 453 |
| **RMS (bonds) ††** | 0.008 | 0.009 | 0.009 | 0.008 | 0.008 | 0.008 | 0.008 | 0.008 |
| **RMS (angles) ††** | 1.09 | 1.10 | 1.20 | 1.06 | 1.18 | 1.10 | 1.10 | 1.15 |
| **Ramachandran favored (%)** | 98 | 97 | 96 | 98 | 98 | 98 | 97 | 98 |
| **Ramachandran outliers (%)** | 0 | 0.22 | 0.44 | 0 | 0 | 0 | 0.44 | 0.22 |
| **Clashscore** | 5.14 | 4.12 | 5.93 | 3.74 | 4.12 | 5.98 | 4.25 | 7.58 |
| **Average B-factor (^2^)** | 44.80 | 50.10 | 65.30 | 44.40 | 48.10 | 43.40 | 43.50 | 48.30 |
| **macromolecules** | 43.20 | 50.00 | 65.20 | 43.30 | 47.80 | 42.80 | 42.30 | 47.10 |
| **ligands** | 47.90 | 53.80 | 77.10 | 49.70 | 54.40 | 49.40 | 47.70 | 54.50 |
| **solvent** | 56.00 | 50.90 | 56.20 | 53.50 | 56.70 | 48.80 | 53.70 | 57.80 |

Statistics for the highest-resolution shell are shown in parentheses.

^†^ *R* _merge_ =
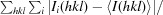

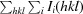
, where *I_i_*(*hkl*) is the intensity of the *i*th measurement of reflection *hkl* and *I*(*hkl*) is the mean value of *I_i_*(*hkl*) for all *i* measurements.

^‡^ *R* _work_ =
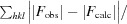

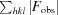
, where *F* _obs_ is the observed structure factor and *F* _calc_ is the calculated structure factor.

^§^ *R* _free_ is the same as *R* _cryst_ except calculated with a subset (5%) of data that were excluded from the refinement calculations.

^††^Engh Huber (1991).
